# Supplementary material for: Usnic Acid and Usnea barbata (L.) F.H. Wigg. Dry Extracts Promote Apoptosis and DNA Damage in Human Blood Cells through Enhancing ROS Levels
Source: Antioxidants (Basel). 2021 Jul 23;10(8):1171. doi: 10.3390/antiox10081171 (PMC8388874; doi:10.3390/antiox10081171)
Supplement: Supplementary file 1 [file antioxidants-10-01171-s001.zip › Supplementary Material - Additional data for semi-preparative UHPLC method .pdf]

Usnic acid and *Usnea barbata* (L.) F. H. Wigg. Dry Extracts promote Apoptosis and DNA Damage in Human Blood Cells through Enhancing ROS Levels

Violeta Popovici [1], Elena Matei [2,*], Georgeta Camelia Cozaru [2, 3], Mariana Aschie [2, 3], Laura Bucur [4,*], Dan Rambu [5], Teodor Costache [5], Iulia Elena Cucolea [5], Gabriela Vochita [6], Daniela Gherghel [6], Aureliana Caraiane [7], Victoria Badea [1]

Additional data for semi-preparative UHPLC method (2.1.1.)

The changes consisted of increasing the critical parameters value and the size of the chromatographic column, injection volume, concentration, and mobile phase flow. The PerkinElmer® Flexar® FX-15 UHPLC system equipped with a Flexar FX PDA-Plus photodiode array detector and a Cosmosil 5-C18-AR-2 chromatographic column, with a length of 150 mm and an inner diameter of 20 mm (producer: Nacalai Tesque, Japan) were used. Technical changes consisted of inserting an injection loop of 400 μL connected between the pump and the column and a manual injection valve to facilitate injection of the sample in high volumes. In addition, a T-splitter was inserted behind the column. Its role was to divert the sample flow to the detector (around 1 ml) and Gilson FC 203B fraction collector. Furthermore, the path length from the splitter to the fraction collector was optimised to adjust the detector flow.

The samples were prepared at concentrations of 1.25 mg/mL, 3 mg/mL, 8 mg/mL and 10 mg/mL to test the maximum injection capacity. The retention time of the usnic acid is reported around 13 min, at a flow rate of 10 mL/min. Therefore, the sample concentrations that could be injected without peak-splitting phenomena were 3 mg/mL at 282 nm and 8 mg/mL at 254 nm. It is known that usnic acid has a maximum absorption at 282 nm. Also, it was observed that running the analysis at that wavelength can surpass the detector higher limit when injecting more concentrated samples. Thus, setting the analytical wavelength to a lower one would improve peak shape and visualisation to assist better isolation at higher concentrations.
